# Balancing Function and Proprioception: A Case Report Featuring Computer-Aided Design/Computer-Aided Manufacturing (CAD/CAM)-Fabricated Marburg Denture System

**DOI:** 10.7759/cureus.69006

**Published:** 2024-09-09

**Authors:** Ritul Jain, Sweta G Pisulkar, Seema R Kambala, Akansha Bansod, Arushi Beri, Shruti Deshmukh

**Affiliations:** 1 Department of Prosthodontics, Crown and Bridge, Sharad Pawar Dental College and Hospital Datta Meghe Institute of Higher Education and Research, Wardha, IND

**Keywords:** cad cam, double crown system, marburg denture, proprioception, selective laser sintering

## Abstract

The ultimate purpose of a partial prosthetic denture is to protect the rest of the teeth as their discarded functionality is being put back together. A Marburg double crown is a form of retainer that works well by providing splinting action between many abutment teeth as well as retention and support. Compared to traditional clasp-retained removable partial dentures (RPDs), the Marburg double crown transfers load to the abutment teeth's long axis. The Marburg double crown system fabricated efficiently using the computer-aided design/computer-aided manufacturing (CAD/CAM) system for treating partially edentulous patients is highlighted in this case report.

## Introduction

“Perpetual preservation of what remains is more important than the meticulous replacement of what is missing.” This is a very insightful statement by DeVan, and no dental community in the world has ever disputed or disapproved of this [1]. To obtain the best possible functionality and aesthetics, every attempt should be made to preserve the natural teeth during the restoration process. Tooth loss has been associated with appetite reduction and nutritional value loss. Therefore, improving whole or just somewhat dentulous circumstances leads to an enhancement of the masticatory system's functionality [2]. Retaining removable partial dentures (RPDs) may be accomplished using an efficient telescopic or double crown system. It offers direction, support, stability, and resistance against movements that may tip the denture off. Additionally, it disperses force along the abutment teeth's long axis [3].

Compared to traditional clasp-retained RPDs, the double crown method exhibits better denture retention and occlusal load transmission to the abutment teeth's long axis [4]. The outer telescope, sometimes known as the secondary crown, and the inner sleeve coping make up the double crown retainer [5]. The force that lifts the prosthesis from its soft-tissue-supported region is the root's long axis because of the circumferential proximity of the secondary crown to the tooth as its abutment [6]. Double crowns are made up of an inner main coping and an external secondary coping, respectively. The primary coping that is bonded onto the abutment teeth shields them from heat irritants and decay. Furthermore, it provides the secondary copings with stiffness and retention, enabling them to cooperate with the inner coping to form a telescoping unit that functions as a similar support for the remaining teeth [7].

As stated, the Marburg denture tensions are moved from the roots' periodontal ligament towards the bone of that alveolus. As the input from proprioception of the periodontal ligament limits occlusal loading, It, therefore, stops the residual ridge that encircles the foundation from resorbing. Unlike conventionally removed partial dentures, they provide more adaptability. Regarding biting power, the effectiveness of chewing, and even phonetics, is enhanced [8]. With the advancement of new techniques and computer-aided design/computer-aided manufacturing (CAD/CAM) technology, metal processing is now accessible. These techniques include selective laser sintering, melting, and grinding.

Milled crowns have a marginal and internal fit that is either superior to or comparable to that of cast crowns [9-11]. Additionally, studies about the resistance to corrosion and fracture have shown that milled specimens perform better [12-13]. Primary crowns made using CAD/CAM technology are now often used in double-crown-retained RPDs. By contrast, CAD/CAM technology is seldom utilized in the creation of secondary crowns and denture frameworks. However, by helping to avoid casting errors and corrections by a specialist, this technology could make the production of this type of denture easier. The number of dental technicians will probably decline [14]. This case report explores the fabrication and design of a Marburg denture in a stepwise procedure with CAD/CAM.

## Case presentation

A 71-year-old male with a chief complaint of trouble with mastication presented to the Department of Prosthodontics. The patient's medical history was remarkable for type 2 diabetes for which he was on medication. Upon intraoral examination, the patient had maxillary left and right molars and anterior teeth and premolars were missing., i.e., 16, 17, 18, 26, 27, and 28 teeth were present. On clinical and radiographic examination, it was observed that 18 and 28 were periodontically compromised and a fixed partial denture with 43-35, and that 44-47 and 36-37 were missing.

It was determined that prosthodontic rehabilitation of both the maxillary and mandibular teeth should be done based on the clinical circumstances and the patient's desires. The patient's reluctance to undergo any surgical intervention meant that implant-supported prostheses were not a viable therapy option. The only beneficial course of treatment was a tooth-supported removable partial denture, given his age and medical history. The patient was counseled about the advantages and disadvantages of each potential therapy option. After taking into account the patient's preferences, compliance level, and the opinions of the physician, it was decided to use fixed removable prostheses for prosthodontic rehabilitation of the maxillary and cast partial denture with a mandibular ridge.

**Figure 1:**
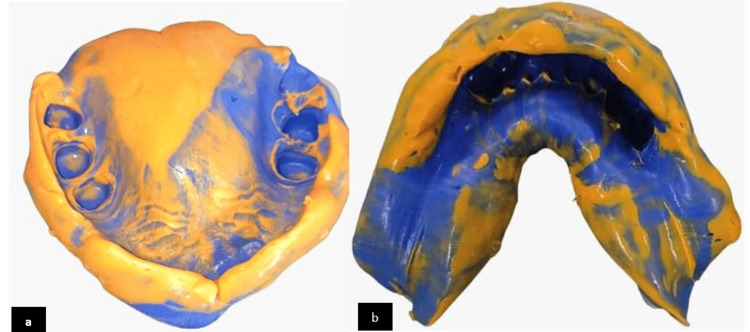
Images of final impression a. Maxillary. b. Mandibular

Following a primary impression, casts for diagnosis were created, surveyed virtually, and examined. Additionally, it was determined that the patient would get a dual-crown structure featuring a clearance fit. The abutment teeth were prepared to be able to receive the telescopic crowns. The thin-cast coping used for telescopic or inner crowns was luted to the abutment teeth. The way to cope was only created parallel to the outer crown in the apical third. This was done to give the clearance fit. The final impression was made for the maxillary Marburg denture system upon luted abutment teeth with primary coping and for mandibular distal extension cast partial denture (Figure 1).

**Figure 2:**
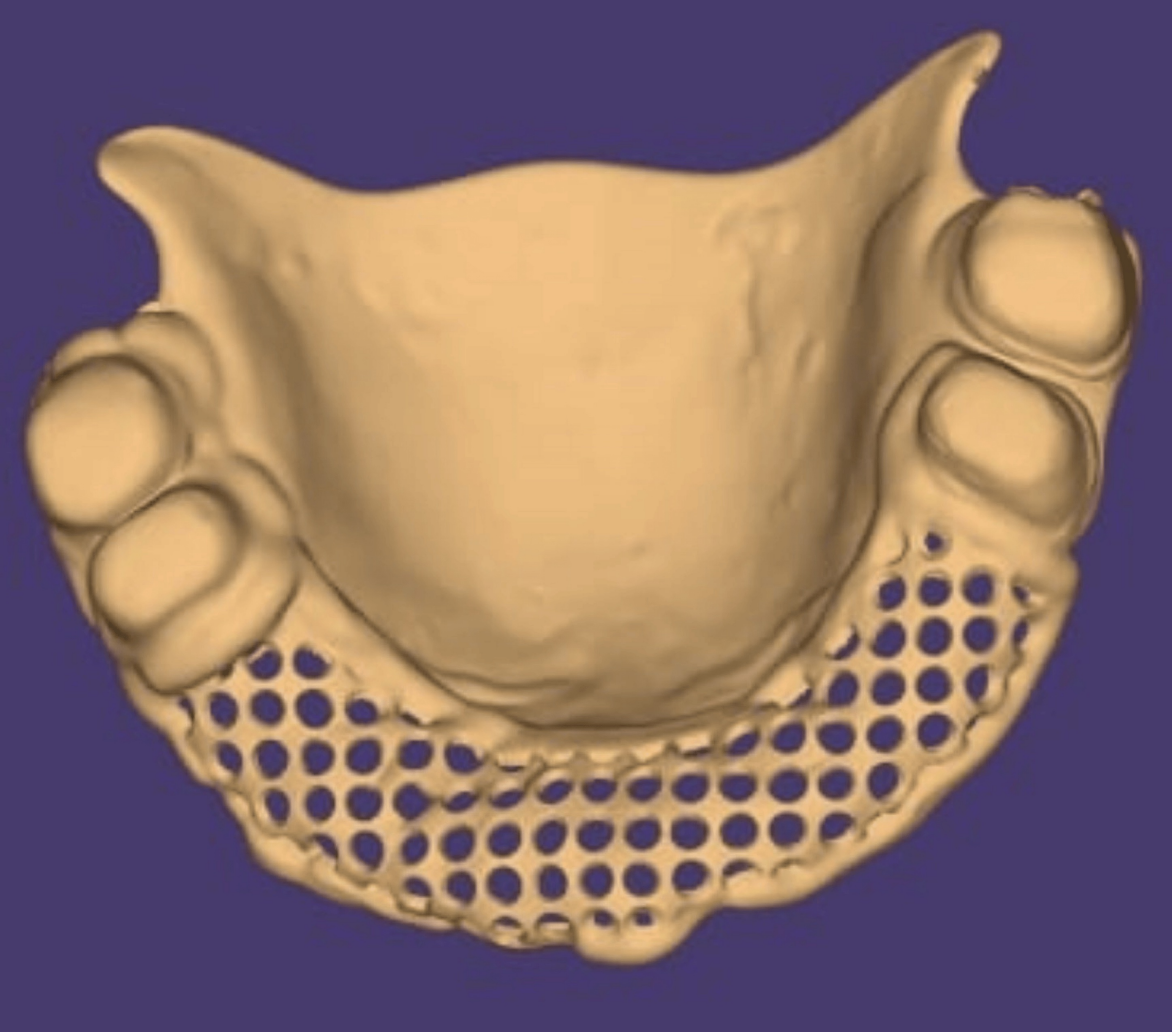
The designing of maxillary framework through exocad software

**Figure 3:**
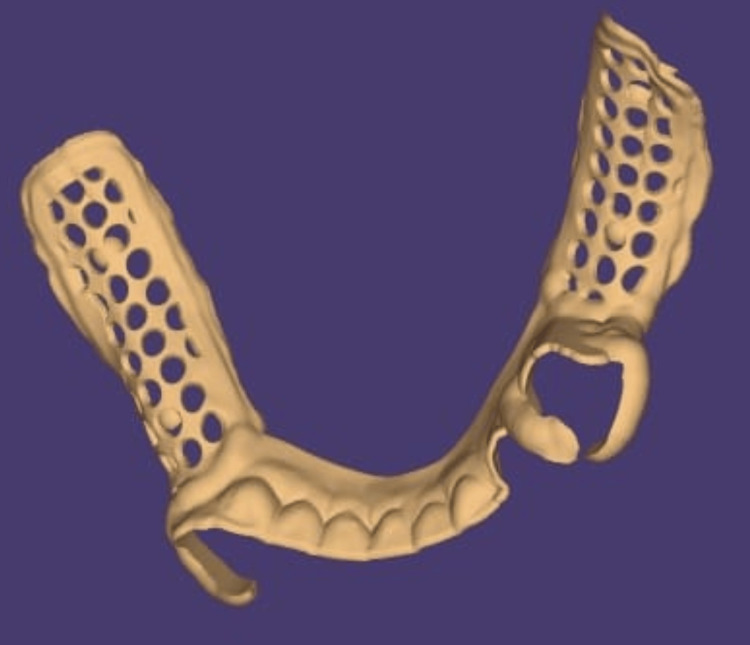
The designing of mandibular framework through exocad software

Both the inner and outer crowns might be accommodated with sufficient space. The cast was then scanned with the CAD system, and a framework was designed for the same (Figures 2-3).

**Figure 4:**
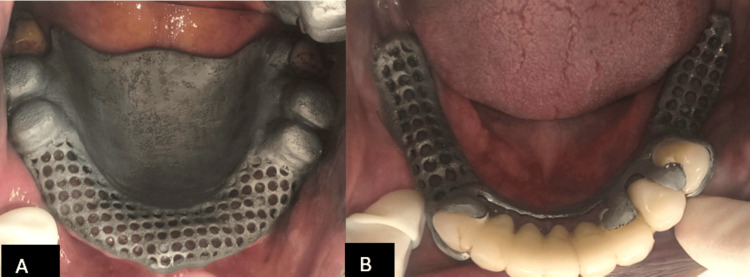
Try-in of maxillary and mandibular metal framework

The denture's structure and outer crowns were fabricated with a CAD/CAM system and cast in a complete Cr-Mo alloy. To confirm the fit, the cast framework was placed into the patient's mouth (Figure 4).

**Figure 5:**
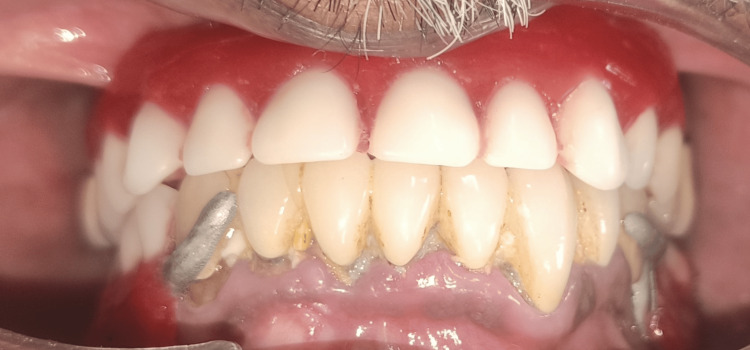
Wax try-in

Without any wedging or friction, the outer crowns fit neatly onto the inner crown. This clearance fit allowed for smooth sliding throughout the insertion route's long axis and very little, undetectable lateral movement. Following the metal try-in, the acrylic shade was chosen. After the metal try-in, a jaw relation was recorded following the recording of the jaw relations. The ability to make simple occlusal modifications was one benefit of having acrylic teeth. The occlusion, aesthetics, and phonetics were all properly assessed during the wax try-in (Figure 5).

**Figure 6:**
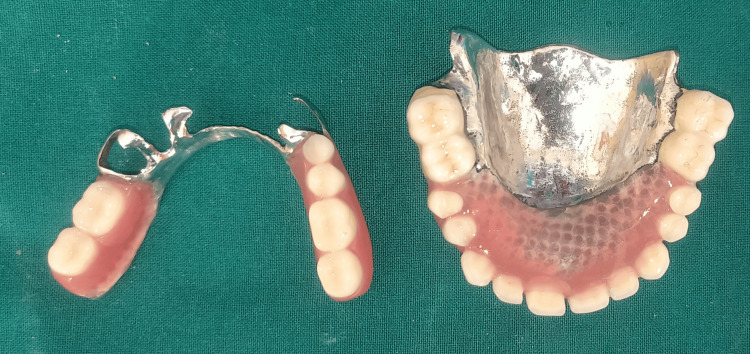
Final finished and polished denture

**Figure 7:**
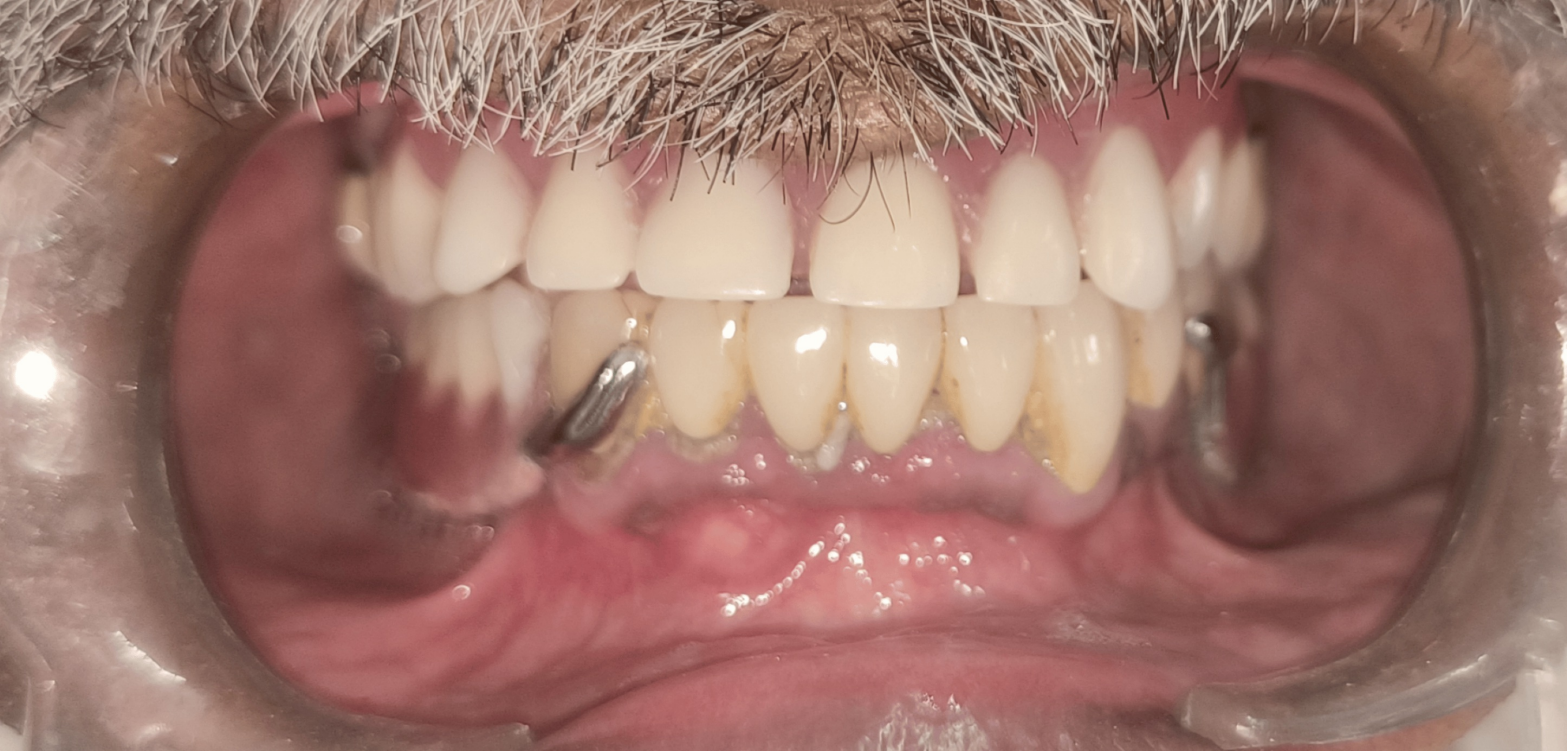
Final prosthesis after insertion

The marginal periodontium of the abutment teeth was exposed by the denture base. After being acquired, finished, and polished, the dentures were placed and assessed (Figures 6-7).

**Figure 8:**
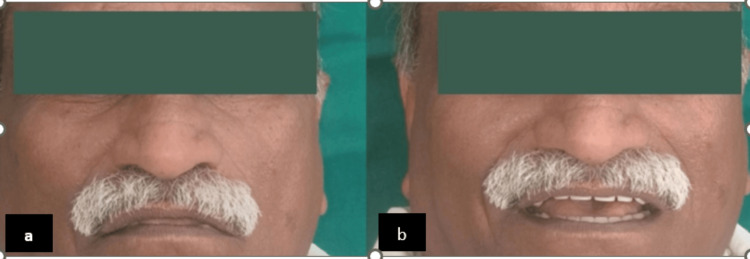
Comparison of pre and postoperative images

Once more, phonetics, aesthetics, and fit occlusion were assessed. Post-insertion guidelines were given and maintaining good hygiene was stressed. Figure 8 shows a comparison of pre and postoperative pictures.

## Discussion

RPD's longevity depends on the kind of double crown system retention mechanism. The main goal of twin crowns in RPD is to reduce the damaging occlusal stresses that are weaving, horizontal, and directed more axially. Telescopic or double crown enables numerous abutment and cross-arch splinting [15]. The Marburg double crown system was initially described by Lehmann and Gente. It is a dynamic technique for replacing partially missing dental arches, using implants or natural teeth as abutments. Its application is unaffected by the quantity and placement between the abutments. The denture base does not cover the abutment teeth's marginal periodontium. They were modified in 1980 by Langer, who divided them into three systems: inner crowns that are cylindrical, resilient crowns, and conical crowns [16,17]. Ishida et al. noted a survival rate of 100% for telescopic-retained RPD vs. 94.5% for conventional RPD. Traditional RPD patients often report higher rates of caries and periodontitis, albeit with no statistically significant difference [18].

CAD/CAM technologies have been successfully used to construct a telescoping denture's RPD framework SLM using Ti-6Al-4V and PEEK frameworks for a complete mouth rehabilitation case [19]. In the case study, the Marburg denture, which was made with CAD/CAM technology, showed remarkable fit and aesthetics. Research has repeatedly demonstrated that CAD/CAM dentures fit better than dentures made with conventional procedures. This is because it is now possible to correct manual errors and fine-tune designs digitally before manufacturing. In addition, the Marburg design improves stability and aesthetics compared to conventional denture designs by incorporating functional elements and anatomical markers into the denture.

The CAD/CAM technology offers both advantages and drawbacks in terms of production. A few problems involving software and materials, as well as differentiation between digital design and clinical results have been reported. Ongoing modifications in CAD/CAM devices have overcome one of the numerous challenges faced in this industry. Such advances help to ensure that processing is done quickly and flawlessly through both hardware and software.

## Conclusions

Our report shows that CAD/CAM technology can truly revolutionize the field of prosthodontics, by crafting precious alloys, such as those used in the creation of Marburg dentures. Compared to conventional clinical rehabilitation, the exact fit and adaptability of the CAD/CAM technology is another advantage of the latest rehabilitation procedures that include computer-aided design and milling, which are automated. However, there are certain obstacles regarding CAD/CAM-fabricated teeth, such as those related to the selection of material or the navigation of software. However, these problems are being addressed by continuous improvements, which help make CAD/CAM denturing a more reliable and widely available treatment option.
